# Evaluation of the distance from the anterior cervicovaginal junction to the anterior peritoneal reflection for anterior colpotomy during vaginal hysterectomy in Korean women

**DOI:** 10.1097/MD.0000000000026941

**Published:** 2021-08-20

**Authors:** Ji Hye Lee, Su Hyun Chae, A. Jin Lee, Yoon Jung Min, Kyeong A. So, Sun Joo Lee, Tae Jin Kim, Seung-Hyuk Shim

**Affiliations:** a Department of Obstetrics and Gynecology, Research Institute of Medical Science, Konkuk University School of Medicine, Seoul, Korea; b Department of Obstetrics and Gynecology, Korea University Anam Hospital, Korea University College of Medicine, Seoul, Korea.

**Keywords:** cervicovaginal incision, laparoscopically assisted vaginal hysterectomy, peritoneal reflection, vaginal hysterectomy

## Abstract

This study aimed to know the distance of the cervicovaginal junction (CVJ) to the anterior peritoneal reflection (APR) as measured in surgical specimens, and assess the distance between the CVJ and APR to ensure safe anterior colpotomy for vaginal hysterectomy among Korean women.

Patients who underwent vaginal hysterectomy were included in the analysis. According to the presence of pelvic organ prolapse or menopausal status, the distance from the CVJ to the APR was assessed preoperatively through transvaginal ultrasonography (TV-US), as well as intraoperatively using surgical specimens. The intraclass correlation coefficient was used to determine the reliability between 2 measurements.

In total, 171 patients were included. The median distance from the CVJ to the APR measured on TV-US was 19.8 (3.3–41.3) mm. Meanwhile, the median distance from the CVJ to the APR measured using the surgical specimen was 26.0 (12.0–55.0) mm. The intraclass correlation coefficient for the absolute agreement between 2 measurements was 0.353 (95% confidence interval: 0.002–0.570; *P* < .001), which is indicative of poor reliability. The median distance from the CVJ to the APR measured using the surgical specimen did not differ significantly between the 2 groups according to pelvic organ prolapse (26.0 [12.0–55.0] vs 27.5 [17.0–55.0] mm, *P* = .076] and menopausal status (27.0 [15.0–55.0] vs 26.0 [12.0–55.0] mm, *P* = .237).

TV-US does not an accurately measure the dissection plane length from the CVJ to the APR during anterior colpotomy. During vaginal hysterectomy, the median distance from the CVJ to the APR measured using the surgical specimen was 26 (12.0–55.0) mm, which can help decrease surgical complications.

## 1 Introduction

Hysterectomy is among the most commonly performed surgical procedures in the gynecologic field.^[1]^ In the United States, approximately 600,000 women undergo this procedure annually.^[2]^ In Korea, 43,725 women underwent hysterectomy in 2017, and according to the health data of the Organization for Economic Cooperation and Development, the prevalence of hysterectomy per 100,000 women was 170.4 in 2017.^[3]^ The common indications for hysterectomy include leiomyoma, abnormal uterine bleeding, pelvic pain, and pelvic organ prolapse. Hysterectomy can be performed using several approaches, including abdominal or minimally invasive techniques (vaginal, laparoscopic, robotic, or Transvaginal natural orifice transluminal endoscopic surgery (vNOTES)). If feasible, hysterectomy using minimally invasive techniques should be performed because it has well-established advantages compared with abdominal hysterectomy.^[2]^

Vaginal hysterectomy is the oldest known minimally invasive procedure, and it has better surgical outcomes than abdominal hysterectomy.^[4]^ The use of this approach is decreasing due to insufficient training among surgeons, diversity of operative approaches, and several technically challenging steps of this procedure.^[5]^ In particular, the anterior colpotomy technique is required to enter the anterior cul-de-sac (ACDS). This procedure is important in performing not only vaginal hysterectomy but also vNOTES and laparoscopically assisted vaginal hysterectomy. After a circumferential incision is made at the level of the anterior cervicovaginal junction (CVJ), surgeons dissect the overlying vaginal mucosal layer off the underlying cervical stroma until the level of the peritoneal reflection of the ACDS. This is a blind technique; thus, surgeons have performed the procedure bluntly based on their individual experiences, and some patients presented with bleeding or bladder injury.^[6]^ Information about surgical anatomy, including the distance of the anterior CVJ to the anterior peritoneal reflection (APR) measured from the surgical specimen, is essential to ensure safe anterior colpotomy. However, there is a lack of data on the distance from the incision site of the anterior CVJ to the lower pole of the anterior peritoneal reflection (APR), which comprise the vesicocervical and vesicouterine spaces. Therefore, the current study aimed to evaluate the distance of the dissection plane from the anterior CVJ to the APR during vaginal hysterectomy among Korean women.

## 2 Materials and methods

**Figure 1:**
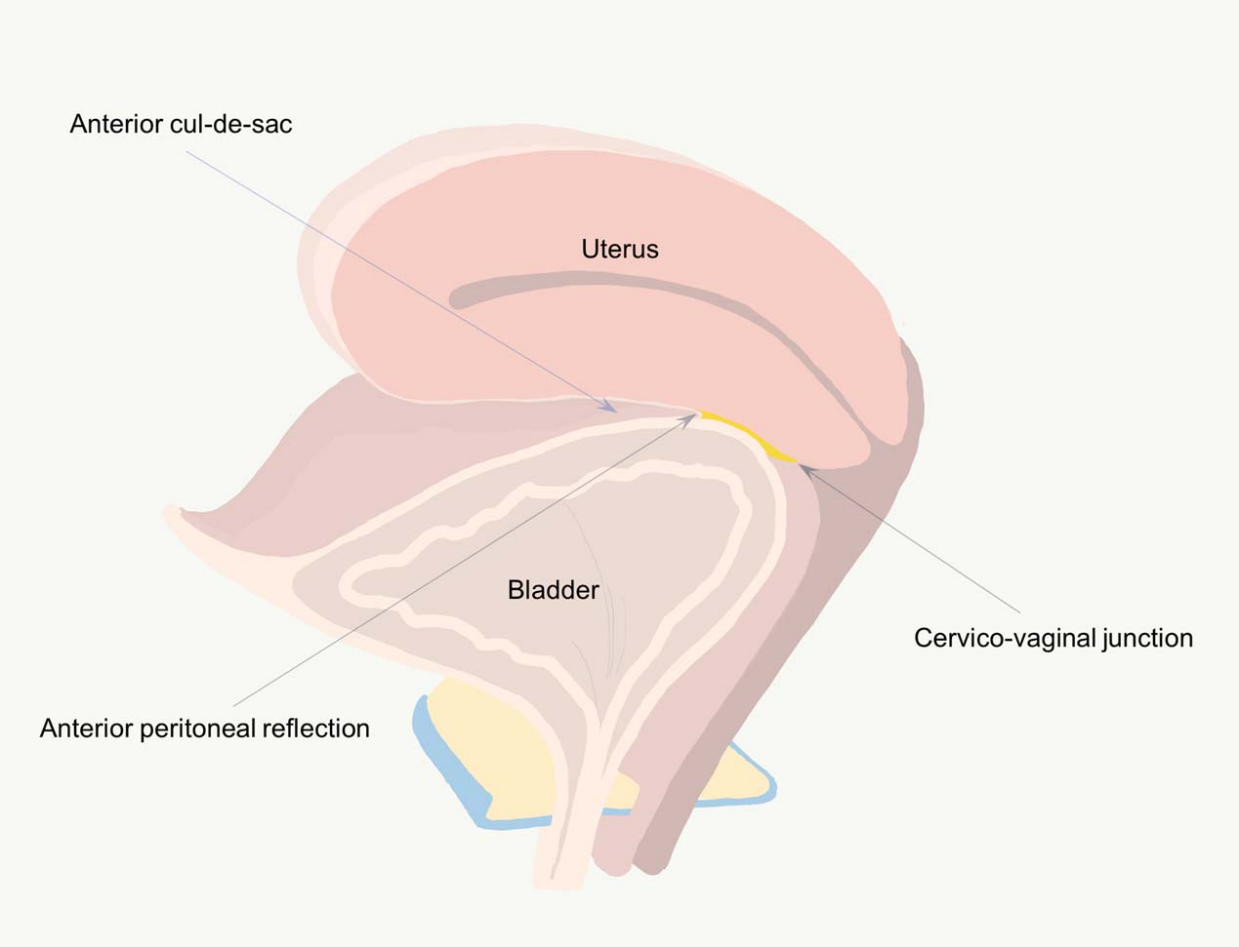
Schematic drawing of the pelvis showing the bladder, uterus, anterior cul-de-sac, cervicovaginal junction, and anterior peritoneal reflection.

Figure 1 shows schematic drawing of the pelvis showing the bladder, uterus, anterior cul-de-sac, CVJ, and APR. To examine the distance and dissection plane from the CVJ to the APR, our prospectively maintained database of patients who underwent vaginal hysterectomy was retrospectively reviewed from September 2016 to August 2019. Patients who underwent vaginal hysterectomy for benign diseases, such as leiomyoma, adenomyosis, abnormal uterine bleeding, pelvic organ prolapse, or premalignant lesions, including cervical intraepithelial neoplasia, were included. Meanwhile, patients with a history of cesarean delivery, any previous surgeries affecting this region, any lesions causing significant anatomic distortion of this region, and gynecologic malignancy that may cause difficulties in measuring the distance from the CVJ to the APR were excluded. The institutional review board of our institution (KUH1040065) approved the study, and the patients provided informed consent.

**Figure 2:**
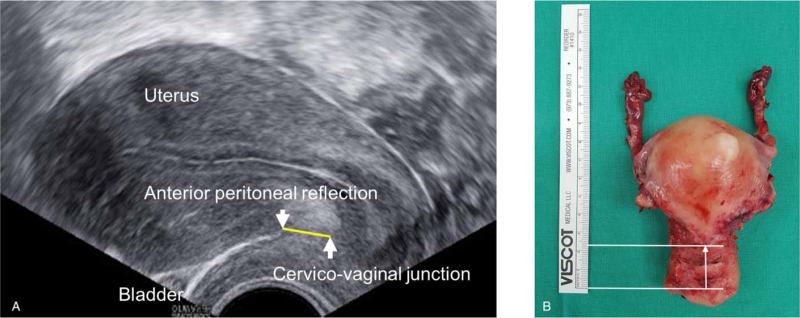
A, Distance from the cervicovaginal junction to the anterior peritoneal reflection measured on transvaginal ultrasonography. B, Distance from the cervicovaginal junction to the anterior peritoneal reflection measured using the surgical specimen. A white line indicates the distance measured from the cervicovaginal junction to the lower limit of the uterine serosa.

All patients underwent preoperative transvaginal ultrasonography (TV-US) within 1 month of the surgery date. The patient was instructed to void, and a complete transvaginal sonogram was performed. Physicians or sonographers who specialize in TV-US measured the distance from the CVJ to the APR as well as the size, version, and shape of the uterus, adnexa, and adjacent organs (Fig. 2A).

The bladder was drained before anterior colpotomy. When the cervix was grasped and pulled downward with 2 tenacula, the CVJ could be easily identified using the decreasing rugae or the transverse fold mark between the movable vaginal wall and the smooth cervical tissue. Initial incision for anterior colpotomy may vary among surgeons and this could bias the measurement of the distance. To minimize variability and ensure consistent measurement of the anatomical distance from the CVJ to the APR, the initial circumferential incision for anterior colpotomy was made at the level of the CVJ using a knife or electrosurgical instrument as introduced in the textbook.^[7]^ Once the appropriate depth of the incision was achieved, the vaginal tissue falls away from the underlying cervical tissue via sharp dissection with the scissors. Vesicocervical and vesicouterine spaces were further developed, thereby eventually allowing access to the APR. Then, the APR was grasped and incised, which led to the ACDS. After the completion of anterior colpotomy, posterior colpotomy was performed. The uterosacral and cardinal ligaments were consecutively treated with complete vaginal hysterectomy. After the surgical removal of the uterus, the distance from the CVJ to the incision site of the APR, which is the lower margin of the uterine serosa,^[8]^ was measured using a flexible 15-cm ruler (Fig. 2B). The uterine weight was measured right after the surgical removal of the uterus.

Clinicopathological data, such as those of age, body mass index (BMI), parity, menopausal status, indication for surgery, surgical procedure name, distance from the CVJ to the APR measured on TV-US, distance from the CVJ to the APR measured using the surgical specimen, and uterine weight, were collected from the electronic medical records. BMI was measured within 7 days of the surgery date.

Based on the normality of the distribution of continuous variables, the Student *t* test or the Mann–Whitney *U* test was used to compare the mean values between the 2 groups. The intraclass correlation coefficient was used to quantify the proportion of variance between the distances from the CVJ to the APR measured on TV-US and using the surgical specimen. *P* values < .5 indicated poor reliability; *P* values of .5 ≤ values < .75, moderate reliability; *P* values .75 ≤ values < .90, good reliability; and *P* values ≥.90, excellent reliability.^[9]^ A statistical analysis was performed using the Statistical Package for the Social Sciences software package version 25.0 (Chicago, IL), and a *P* value < .05 was considered significant.

## 3 Results

During the study period, a total of 171 patients were included. The baseline characteristics of the participants are summarized in Table 1. The median age was 56 (range: 37–83) years, and there were a total of 103 (59.6%) postmenopausal women. The most common surgical indication was myoma and/or adenomyosis (40.4%), followed by premalignant lesion of cervix (24.6%), pelvic organ prolapse (24.6%), and endometrial pathologies (9.9%). The median distance from the CVJ to the APR measured on TV-US was 19.8 (range: 3.3–41.3) mm. Meanwhile, the median distance from the CVJ to the APR measured using the surgical specimen was 26.0 (range: 12.0–55.0) mm. The intraclass correlation coefficient for the absolute agreement between 2 measurements was 0.353 (95% confidence interval: 0.002–0.570; *P* < .001), which is indicative of poor reliability. None of the patients presented with urinary tract injury during hysterectomy procedure.

**Table 1:** Clinicodemographic and pathological characteristics of the participants.

	Total (n = 171)
Age, yr, median (range)	56 (37–83)
BMI, kg/m^2^, median (range)	23.7 (16.1–39.6)
Premenopausal, n	68 (40.4%)
Postmenopausal, n	103 (59.6%)
Parity, n, median (range)	2 (0–6)
Indication for hysterectomy, n	
Myoma and/or adenomyosis	69 (40.4%)
Premalignant lesion of cervix	42 (24.6%)
Pelvic organ prolapse	42 (24.6%)
Endometrial pathology	17 (9.9%)
The distance from cervix to fundal apex by TV-US, mm, median (range)	67.0 (29.1–140.0)
The distance from CVJ to APR measured by TV-US, mm, median (range)	19.8 (3.3–41.3)
The distance from CVJ to APR measured from specimen, mm, median (range)	26.0 (12.0–55.0)
The weight of uterus, gram, median (range)	98.3 (13.0–686.0)

APR = anterior peritoneal reflection, BMI = body mass index, CVJ = cervicovaginal junction, TVU = transvaginal ultrasonography.

Table 2 shows the measurements between the 2 groups according to pelvic organ prolapse. Age, parity, and uterine weight differed significantly between the 2 groups. However, the median distance from the CVJ to the APR measured on TV-US did not differ significantly between the 2 groups according to pelvic organ prolapse (21.2 [range: 3.3–46.1] vs 17.7 mm [range, 5.4–40.6], *P* = .083). In addition, the median distance from the CVJ to the APR measured using the surgical specimen did not differ significantly between the 2 groups (26.0 [range: 12.0–55.0] vs 27.5 mm [range, 17.0–55.0], *P* = .076). The intraclass correlation coefficient for the absolute agreement between 2 measurements in the group with pelvic organ prolapse was 0.348 (95% confidence interval: −0.201–0.665; *P* < .05), indicating poor reliability.

**Table 2:** Comparison of clinicodemographic characteristics and measurements according to pelvic organ prolapse.

	Without pelvic organ prolapse (n = 129)	With pelvic organ prolapse (n = 42)	*P* ^∗^
Age, yr, median (range)	53 (37–83)	68 (50–83)	**< .05**
BMI, kg/m^2^, median (range)	23.5 (18.1–35.3)	24.3 (16.1–39.6)	.209
Parity, n, median (range)	2 (0–6)	3 (2–6)	**< .05**
The distance from CVJ to APR measured by TV-U, mm, median (range)	21.2 (3.3–46.1)	17.7 (5.4–40.6)	.083
The distance from CVJ to APR measured from specimen, mm, median (range)	26.0 (12.0–55.0)	27.5 (17.0–55.0)	.076
The weight of uterus, gram, median (range)	143.5 (13.0–686.0)	59.5 (26.5–341.0)	**< .05**

APR = anterior peritoneal reflection, BMI = body mass index, CVJ = cervicovaginal junction, TVU = transvaginal ultrasound.

∗ Mann–Whitney *U* test. The bold values mean significant *P* values.

Table 3 shows the measurements between the 2 groups according to menopausal status. Age, BMI, parity, and uterine weight differed significantly between the 2 groups. The median distance from the CVJ to the APR measured on TV-US differed significantly between the 2 groups according to menopausal status (23.7 [range: 12.0–46.1] vs 17.2 mm [range, 3.3–35.1], *P* < .05). However, the median distance from the CVJ to the APR measured using the surgical specimen did not significantly differ between the 2 groups (27.0 [range: 15.0–55.0] vs 26.0 mm [range: 12.0–55.0], *P* = .237). In both premenopausal and postmenopausal groups, the intraclass correlation coefficient for the absolute agreement between the 2 measurements was 0.371 (95% confidence interval: 0.016–0.603; *P* < .05) and 0.296 (95% confidence interval: −0.125–0.561; *P* < .05), respectively, indicating poor reliability.

**Table 3:** Comparison of clinicodemographic characteristics and measurements according to menopausal status.

	Premenopausal (n = 68)	Postmenopausal (n = 103)	*P* ^∗^
Age, yr, median (range)	48 (37–54)	64 (46–83)	**< .05**
BMI, kg/m^2^, median (range)	22.8 (18.1–35.3)	24.2 (16.1–39.6)	**< .05**
Parity, n, median (range)	2 (0–3)	2 (0–6)	**< .05**
The distance from CVJ to APR measured by TV-U, mm, median (range)	23.7 (12.0–46.1)	17.2 (3.3–35.1)	**< .05**
The distance from CVJ to APR measured from specimen, mm, median (range)	27.0 (15.0–55.0)	26.0 (12.0–55.0)	.237
The weight of uterus, gram, median (range)	220.5 (42.0–686.0)	68.0 (13.0–545.0)	**< .05**

APR = anterior peritoneal reflection, BMI = body mass index, CVJ = cervicovaginal junction, TVU = transvaginal ultrasound.

∗ Mann–Whitney *U* test. The bold values mean significant *P* values.

## 4 Discussion

Anterior colpotomy is an essential technique used when performing not only vaginal hysterectomy but also other minimally invasive hysterectomies, including laparoscopically assisted vaginal hysterectomy.^[10]^ To minimize the risk of urinary tract injuries or bleeding during anterior colpotomy, one must be knowledgeable about the anatomic structure of the dissection plane to reach the ACDS and adjacent organ structures. The current study showed that the median distance from the CVJ to the APR measured using the surgical specimen was 26 mm. The distance was not significantly different between the 2 groups according to pelvic organ prolapse or menopausal status. This information could be useful for surgeons when performing anterior colpotomy during vaginal hysterectomy or laparoscopically assisted vaginal hysterectomy particularly among Korean women.

Data regarding the distance from the CVJ to the APR in the dissection area of anterior colpotomy during vaginal hysterectomy are limited. In the study by Balgobin et al, the median dissection distance from the initial incision to the peritoneal reflection for anterior colpotomy was 34 (range: 18–46) mm in 22 patients undergoing vaginal hysterectomy.^[8]^ However, the cohort enrolled in that study comprised of individuals of non-Asian ethnicity, and included 12 Hispanic, 5 White, and 2 African American individuals. In addition, the authors acknowledged that one of the limitations of their study is the inclusion of a small number of patients. Meanwhile, the cohort of the present study included mostly Korean women, and the sample size was larger than that of the previous study. The median distance from the CVJ to the APR in our study was shorter than that in previous study, which represents racial differences.

Although the result did not significantly differ, the median distance from the CVJ to the APR was slightly greater in groups with pelvic organ prolapse than in those without. This finding was consistent with that of a previous report.^[8]^ This result might be attributed to the redundancy of pelvic floor tissues or difference in uterine size. However, the result should be interpreted with caution particularly in women with prolapse who present with cervical elongation. Approximately one-third of patients with prolapse experience cervical elongation, which is defined as a cervical length >33.8 mm.^[11]^ Unfortunately, our data could not provide the proportion of patients with prolapse who presented with cervical elongation. Considering that greater distances are explicitly expected with cervical elongation, pelvic organ prolapse quantification evaluation or ultrasonographic evaluation of cervical length could be helpful in estimating the approximate length to the APR in suspected cases of cervical elongation.^[12]^

In the current study, the intraclass correlation coefficient for the absolute agreement between the distances from the CVJ to APR measured on TV-US and using the surgical specimen indicated poor reliability, regardless of pelvic organ prolapse or menopause; therefore, there is no absolute agreement between the 2 measurements. Based on this finding, the distance from the CVJ to the APR measured preoperatively on TV-US might not be used as a surrogate for the length of the dissection plane during anterior colpotomy, implying that the anatomical distance in the surgical specimen can only be identified from vaginal hysterectomy; therefore, the value measured in the surgical specimen in the present study can provide useful information to clinicians performing vaginal hysterectomy. The average distance from the CVJ to the APR measured using the surgical specimen was 6 mm longer than that measured on TV-US. The variations in incision at the CVJ and the stretching effect of uterine traction during surgery might contribute to this result. Moreover, it is often difficult to mark the APR on TV-US because the APR collapses in general as the dome of the bladder abuts the lower segment of the uterus. Thus, routine ultrasonographic evaluation would not be required for the assessment of the distance at this site to enter the ACDS. Nonetheless, in case of difficult anterior colpotomy caused by cervical elongation or previous surgery at this site, ultrasonographic evaluation can provide information for the evaluation of the dissection site and adjacent organ structures.^[12]^

The current study had several limitations. First, we did not measure the distance from the CVJ to the APR in the uterus located in the pelvis before surgical removal. Thus, the distances could not be compared with those measured using the specimen that was surgically removed. Second, the measurements were not estimated by multiple raters at the same time. Thus, the interrater reliability could not be evaluated. As for the power calculation, post-hoc power analyses revealed that a sample size of 171 subjects with 2 observations per subject achieved 76% power to detect an intraclass correlation coefficient of 0.5 under the alternative hypothesis, when the coefficient under the null hypothesis is 0.353 using an *F* test with a significance level of 0.05. Thus, a higher number of patients than that in our study is needed to obtain more than adequate power (i.e., 80%). However, the current study included patients with common clinical indications for vaginal hysterectomy and various demographic conditions, such as pre- or postmenopausal and prolapsed or nonprolapsed uterus, thereby increasing the generalizability of the results for clinical practice.

In conclusion, the median dissection distance from the initial incision at the CVJ to the APR is 26 (12–55) mm. This information could be useful to ensure safe anterior colpotomy to reach the ACDS in Korean women. TV-US does not accurately measure the dissection plane length from the CVJ to the APR during anterior colpotomy. If difficulties are encountered during the dissection of the vesicovaginal and vesicouterine spaces with a significantly longer or shorter distance, the surgical plane should be reevaluated. In such cases, additional techniques, such as bladder transillumination using a cystoscope,^[13]^ posterior colpotomy, and securing the uterosacral and cardinal ligaments, may be utilized. Future large-scale prospective studies are needed to confirm our findings and achieve generalizable results.

## Acknowledgments

This paper was written as part of Konkuk University's research support program for its faculty on sabbatical leave in 2020.

The authors thank Chaewon Shim who made a digital illustration to produce a schematic drawing of the pelvis for this paper.

## Author contributions

**Conceptualization:** Seung-Hyuk Shim.

**Data curation:** Seung-Hyuk Shim, Ji Hye Lee, Su Hyun Chae, A Jin Lee, Yoon Jung Min.

**Formal analysis:** Seung-Hyuk Shim, Su Hyun Chae.

**Investigation:** Seung-Hyuk Shim, Ji Hye Lee, A Jin Lee.

**Methodology:** Seung-Hyuk Shim, Ji Hye Lee, Su Hyun Chae.

**Resources:** Kyeong A So, Sun Joo Lee, Tae Jin Kim, Seung-Hyuk Shim.

**Software:** Seung-Hyuk Shim, Su Hyun Chae.

**Supervision:** Seung-Hyuk Shim.

**Validation:** Kyeong A So, Sun Joo Lee, Tae Jin Kim.

**Visualization:** Su Hyun Chae, Seung-Hyuk Shim.

**Writing – original draft:** Seung-Hyuk Shim, Ji Hye Lee, Su Hyun Chae.

**Writing – review & editing:** Yoon Jung Min, A Jin Lee, Kyeong A So, Sun Joo Lee, Tae Jin Kim, Seung-Hyuk Shim.
